# Quantitative estimation of closed cell porosity in low density ceramic composites using X-ray microtomography

**DOI:** 10.1038/s41598-022-27114-w

**Published:** 2023-01-04

**Authors:** J. D. Smith, C. Garcia, J. Rodriguez, T. W. Scharf

**Affiliations:** 1 Materials Science and Engineering and Advanced Materials and Manufacturing Processes Institute (AMMPI), University of North Texas, Denton, TX 76203 USA; 2 Armor Mechanisms Branch, CCDC Army Research Laboratory, Aberdeen Proving Ground, Aberdeen, MD 21005 USA

**Keywords:** Materials science, Structural materials, Techniques and instrumentation, Theory and computation

## Abstract

X-ray Microtomography is a proven tool for phase fraction analysis of multi-phase systems, provided that each phase is adequately partitioned by some means of data processing. For porosity in materials containing low-density ceramic phases, differentiation between pores and the low-density phase(s) can be intractable due to low scattering in the low-density phase, particularly if small pores necessitate low binning. We present a novel, combined methodology for accurate porosity analysis—despite these shortcomings. A 3-stage process is proposed, consisting of (1) Signal/noise enhancement using non-local means denoising, (2) Phase segmentation using a convolutional neural network, and (3) Quantitative analysis of the resulting 3D pore metrics. This particular combination of denoising and segmentation is robust against the fragmentation of common segmentation algorithms, while avoiding the volitional aspects of model selection associated with histogram fitting. We discuss the procedure applied to ternary phase SiC–TiC-diamond composites produced by reactive spark plasma sintering with porosity spanning 2–9 vol%.

## Introduction

Porosity in ceramics is a regularly unavoidable truth. Whether purposeful, or a natural result of incomplete densification, porosity is directly correlated with reduced mechanical properties, thermal conductivity, and electrical conductivity, among many other things. Contrarily, many applications directly rely upon porosity-induced characteristics, such as acoustic/microwave absorption^1–4^, porosity as a growth surface^3,5^, porosity-mediated flux pinning in high temperature superconductors^6,7^, or thermal insulators^8,9^. Regardless of the origin, porosity characterization is challenging. Basic porosity characterization is usually accomplished by approachable microscopy techniques such as optical or electron microscopy. However, 3D effects like tortuosity, shape, anisotropy, and general connectedness are unobservable by optical/electron microscopies, for which precise quantification is not tenable. Gaseous adsorption methods, like those based on Brunauer–Emmett–Teller (BET) theory, are far more accurate; however, the localized insight is forgone, and closed-cell type porosity is poorly characterized by BET methods.

X-ray Microtomography (XRM) is becoming more common for porosity analysis in ceramics^10–13^. The success of XRM in this regard is predicated on the favorable field of view (number statistics), localization, and indifference towards pore percolation. Complications come in the form of the achievable dynamic range in detectable feature size and the variability of the signal-to-noise ratio (SNR). The bounds of the dynamic range are determined by the detector dimensions (a charge-coupled device), effective pixel size, and objective magnification; the SNR depends on binning, exposure time, and material density/thickness. For porous microstructures dominated by low density phases, the SNR is crucial for viable segmentation results. Conventional segmentation strategies—such as thresholding or watershed—can be overly sensitive to noise, producing numerous false positives if the SNR is sufficiently low. As result, noise reduction is a common step taken to mitigate the effects of poor SNR in all kinds of tomograms. However, all noise reduction algorithms tend to suppress (to a greatly varying degree) the higher spatial frequencies that crucially influence image “sharpness”, or edge-preservation. In the medical and image processing community, this has spurred considerable activity in the pursuit of novel noise reduction algorithms exhibiting speed, edge preservation, approachability (i.e., parameter tuning), and scalability^14,15^. Non-local means (NLM) is one such filter effective in both denoising and edge preservation, already finding widespread use among the greater tomography community^15–18^.

Following noise reduction, segmentation is the step where pores are computationally separated from the image. Segmentation is the general process whereby objective features in an image are isolated, or otherwise mapped for processing. Conventionally, this is achieved through a basic threshold of the scalar distribution, or by application of the watershed. The threshold approach is popularized by its simplicity, requiring only a single parameter for tuning. However, thresholding is susceptible to over-segmentation and is naturally semantic, unable to individualize particles from the resultant mask (without successive processing). The watershed is innately instanced and similarly prone to over-segmentation but is advantaged by the realization of boundaries between contiguous, identical particles^19^.

The advent of the convolutional neural network (CNN) as a segmentation tool has expanded the breadth of classifiable features to those most nebulous in form. Indeed, CNN-based segmentation has seen considerable adoption in the medical community for numerous biological phenomena, for which the prototypical forms of thresholding and watershed are often ill-poised^20^. Image segmentation using CNNs for microscopy purposes has greatly matured since the inception of the CNN, and there are now multiple cloud-based tools for easily leveraging these algorithms, such as APEER (https://www.apeer.com) or CDeep3M^21^. These cloud-based tools reduce the barrier to entry for research groups not directly in the machine learning space, in terms of required technical expertise, computing hardware, and the time commitment for a minimum viable result. Here we use the Zeiss APEER tool to demonstrate that manually constructing CNN architectures for the problem is not strictly required.

In this study we explore the combination of edge-preserving NLM denoising with tailorable convolutional neural network segmentation to analyze porosity in noisy, low contrast XRM experiments. This process is discussed in the context of porous SiC–TiC-diamond composites. Different noise reduction routines are compared to NLM, then the effect of CNN segmentation is contrasted to thresholding for these denoised tomograms. Finally, statistical accuracy is discussed in terms of over/under-segmentation along with error analysis of the porosity volume fraction.

Lastly, this combined methodology is used to investigate the pore evolution in reactively spark plasma sintered SiC–TiC-diamond composites. Resulting from the reactive (liquid-phase) process, XRM reveals a pore morphology representative of the bulk microstructure. Application of the NLM-CNN processing methodology herein allows us to more confidently explicate the competing sintering mechanisms affecting the initial ceramic powder blends. We have recently reported in more detail on these sintering mechanisms in^22^. Furthermore, the combined process presented in this paper has implications for other low-density ceramic composites, such as SiC–B_4_C-diamond composites.

## Materials and methodology

### Materials

Spark plasma sintering (SPS) was used to fabricate intentionally porous SiC and denser SiC–TiC-diamond composites. The SPS system was manufactured by Thermal Technology LLC (Santa Rosa, California). The starting SiC powder (Thermo Fisher Scientific) with D_50_ = 36 µm was sintered at a hold temperature, pressure, time, and heating rate of 1600 °C, 5 MPa, 10 min, and 100 °C/min, respectively. For the composites, powder blends of elemental Si, Ti, and TiC-coated or uncoated diamond were densified using SPS at hold temperatures of 1600, 1625, or 1650 °C at a 10 min hold time with heating rate of 100 °C/min at 50 MPa pressure. Elemental Si and Ti melt during sintering at these temperatures, reacting with diamond to form SiC/TiC. The composite microstructures are primarily SiC-diamond matrix with an intergranular TiC structure (see Fig. 2). Diamond is metastable and tends towards graphitization under these SPS conditions. See^22^ for further composite synthesis and microstructural details.

### X-ray Microtomography

Tomographic images were acquired on a Zeiss XRADIA Versa 520 X-Ray Microscope. The source was configured for 80 kV voltage, 7 W power, and a LE6 filter. Datasets were then acquired at 20 × magnification, 2 × binning (~ 0.797 μm effective pixel size), 7 s exposure time, and − 180°–180° angular stage range.

### Data processing

All XRM experiments are exported from the Zeiss Reconstructor application to uncompressed *.tiff image series in 32-bit floating-point precision, valued between 0 and 1. Image series are reconciled into stacked 3D tiffs (maintaining 32-bit precision) with dimensions 956 × 992 × 964 px using ImageJ^23^. Prior to data processing, the 3D datasets are cropped to 512 × 512 × 512 px cubes positioned at the geometric center of the reconstruction volume (physical dimensions are ~ 408.1 × 408.1 × 408.1 μm or ~ 0.107 mm^3^). Prior to training/segmentation, datasets are downsampled to 8-bit integral precision. Annotation, training, and segmentation are done using APEER (https://www.apeer.com). 3D particle statistics are generated from segmentation results using BoneJ^24^.

## Results and discussion

**Figure 1:**
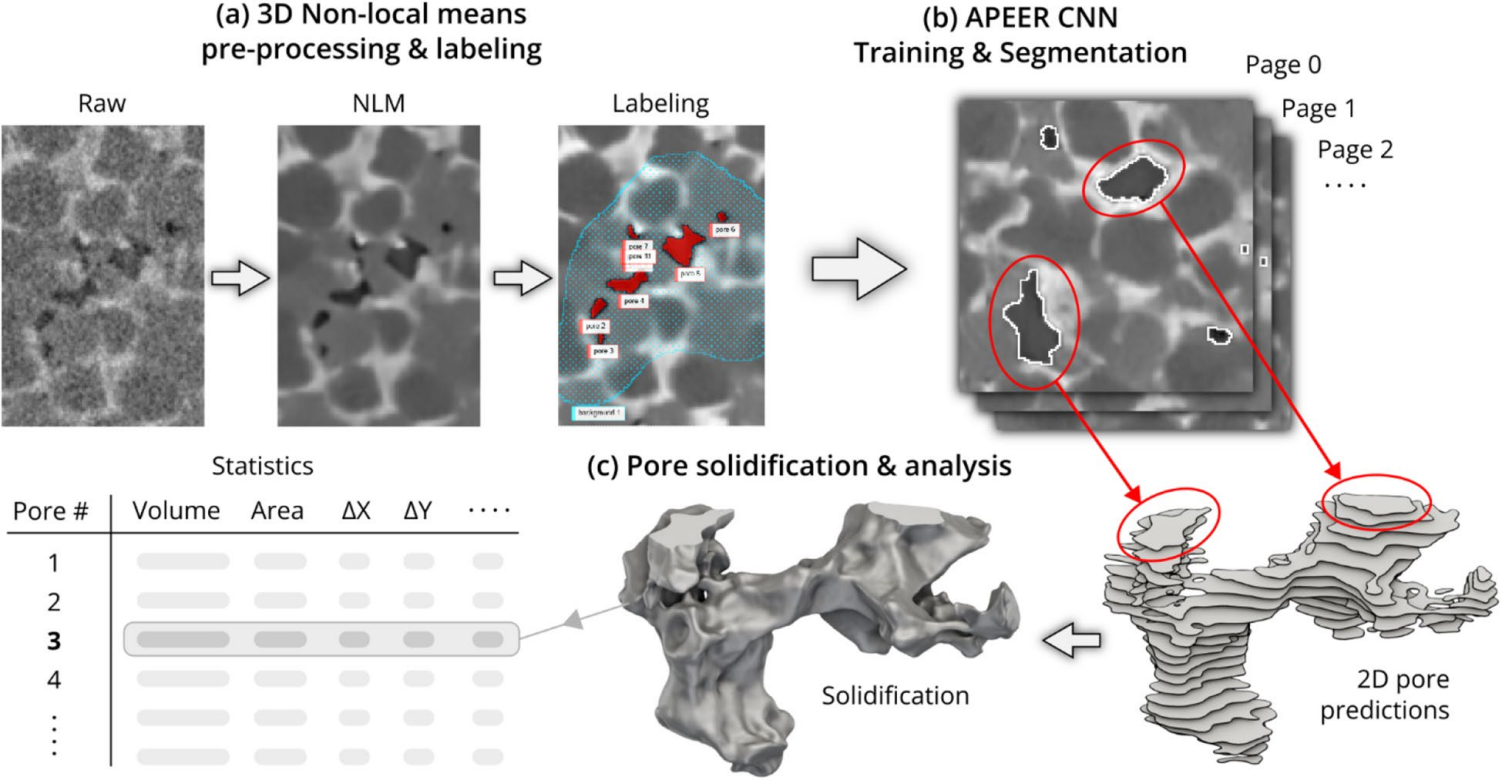
Simplified overview of the entire NLM-CNN analysis process including (**a**) pre-processing and labeling based on non-local means denoising, (**b**) training and segmentation using the APEER convolutional neural network system, and (**c**) reconstruction of the 3D pore structures from 2D predictions and computed statistics. Shown images/slices are cropped to show small collections of pores for clarity, each actual 512 × 512 px slice contains many more pores. Only 26 out of 115 slices in the “skeletal” pore illustration are shown in order to maintain visual clarity.

The proposed process is summarized in Fig. 1, which is composed of 3 main stages. In the first step, 3D non-local means is applied to the raw XRM datasets to improve the SNR. The resulting denoised datasets are used for labeling of pores for training the CNN model in succeeding step. The SNR improvement is intended to have the effect of improving label accuracy by more clearly revealing pores/pore boundaries that are partially obscured by noise, while also reducing the prevalence of noise as a factor which the CNN must “learn”. In the second stage, the labels resulting from annotation of selected 2D slices are used to train a CNN using APEER. More specifically, APEER is a Unet-based convolutional neural network architecture using EfficientNet^25^ as the encoder and Pixel Shuffle^26^ up-scaling within the decoder. Transfer learning is applied by pre-training the decoder on the popular ImageNet^27^ dataset. Prior to training, data augmentation is applied using standard geometric transformations. Once training is completed, the CNN is fed whole XRM datasets for segmentation. The resulting segmentation is two-dimensional in nature, as the CNN is trained on cross-sections of pores. In the third step, the layered 2D segments of each pore are “solidified” back into 3D volumes based on their connected-ness. With the availability of each 3D pore structure, the porosity and other pore-based statistics can be easily measured.

### Denoising

**Figure 2:**
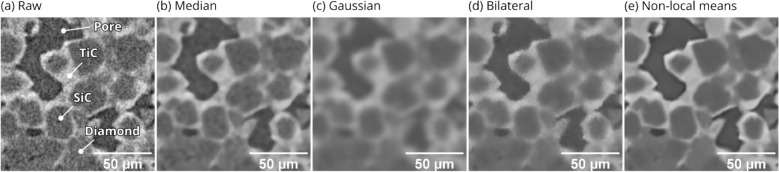
Effect of various denoising filters on a porous SiC-TiC-diamond XRM experiment. All filters are computed in three dimensions (i.e., not paginated). (**a**) Un-processed dataset. (**b**) 5 × 5 × 5 px median filter. (**c**) Gaussian filter with 3 px standard deviation and 4 $\upsigma$ kernel truncation. (**d**) Bilateral filter with gaussian weighting in both spatial and scalar dimensions. The standard deviations are 3 px and 0.2 for the spatial and scalar gaussian weighting, respectively. (**e**) 3D non-local means filter computed with 9 × 9 × 9 px patch size, 7 px search distance, 0.06 standard deviation for weighting, and noise standard deviation estimate of 0.0106.

Improving the signal/noise ratio of an XRM dataset can have a substantial positive effect on the segmentation quality. Unfortunately, denoising algorithms tend to—at least partially—behave like low-pass filters. The suppression of higher spatial frequency features obfuscates vital details like phase boundaries and small particles/pores. As a result, a balance between meaningful noise reduction and the preservation of fine microstructural features is not strictly guaranteed. Figure 2a shows a slice through a porous SiC-TiC-diamond composite, along with various noise reduction filters (Fig. 2b–e). The median/gaussian filters are computed using 3D kernels, and the bilateral/NLM filters using 3D search/patch distances. The gaussian filter is computed with a 3 px standard deviation and the median filter a 5 × 5 × 5 px kernel. Both the median and gaussian filters are simple and fast, requiring only a single parameter for tuning (excepting truncation of the gaussian kernel). The gaussian filter is effective in denoising, at the cost of blurred boundaries and other small features. The histogram peaks belonging to diamond and SiC have partially separated due to the reduction in noise; however, visual clarity in the image has been severely reduced by erasure of higher spatial frequencies. The median filter is effective in the preservation of boundaries and smaller features; however, the achievable level of noise reduction leaves more to be desired. Figure 2d–e shows equivalent results using a bilateral and non-local means filter, respectively. The gaussian, bilateral, and NLM filters are each intimately related, in that each are based on spatial and/or scalar (gaussian) weighted averaging of the neighborhood surrounding each pixel. Given some pixel coordinate ${\varvec{p}}$, the (gaussian) filtered pixel ${\widehat{I}}_{\text{G}}\left({\varvec{p}}\right)$ is the average of each other pixel coordinate ${\varvec{q}}$ in a neighborhood $\Omega$ around ${\varvec{p}}$**,** weighed by a gaussian function ${f}_{\text{G}}$ with standard deviation $\upsigma$:

1 $${\widehat{I}}_{\text{G}}\left({\varvec{p}}\right)={\sum }_{{\varvec{q}}\in\Omega }{f}_{\text{G}}\left(\Vert {\varvec{p}}-{\varvec{q}}\Vert ,\sigma \right){I}_{{\varvec{q}}}$$

where $\Vert {\varvec{p}}-{\varvec{q}}\Vert$ is the spatial distance between pixels ${\varvec{p}}$ and ${\varvec{q}}$. The size of $\Omega$ is usually determined by the number of standard deviations by which the kernel is truncated. As mentioned, the gaussian filter does not have edge-preservation properties. The bilateral filter compensates for this by introducing a second gaussian weighting function ${r}_{\text{G}}$ that weighs against the difference in scalars ${I}_{{\varvec{p}}}$ and ${I}_{{\varvec{q}}}$:

2 $${\widehat{I}}_{\text{BL}}\left({\varvec{p}}\right)=\frac{1}{{C}_{{\varvec{p}}}}{\sum }_{{\varvec{q}}\in\Omega }{f}_{\text{G}}\left(\Vert {\varvec{p}}-{\varvec{q}}\Vert ,\sigma \right){r}_{\text{G}}\left(\left|{I}_{{\varvec{p}}}-{I}_{{\varvec{q}}}\right|,\sigma \right){I}_{{\varvec{q}}}$$

where $1/{C}_{{\varvec{p}}}$ is a normalization factor. This extra weighting function (against scalar distance) allows the bilateral filter to reduce the weight contribution from increasingly dissimilar pixels, giving the bilateral filter moderate edge preservation. This can be seen in Fig. 2d where noise reduction is achieved while maintaining phase boundary clarity. Instead of strictly weighing against spatial or scalar distance, NLM weighs against the similarity of the local neighborhood between ${\varvec{p}}$ and ${\varvec{q}}$^28,29^. This is achieved using a new weighting function $w$, parameterized by the Euclidean distance between each neighborhood (${{\varvec{\Delta}}}_{{\varvec{p}}}$ and ${{\varvec{\Delta}}}_{{\varvec{q}}}$), and a standard deviation $h$, controlling the permissiveness in weighing contributions from dissimilar neighborhoods:

3 $${\widehat{I}}_{\text{NLM}}\left({\varvec{p}}\right)=\frac{1}{{C}_{{\varvec{p}}}}{\sum }_{{\varvec{q}}\in\Omega }w\left({\Vert {{\varvec{\Delta}}}_{{\varvec{p}}}-{{\varvec{\Delta}}}_{{\varvec{q}}}\Vert }^{2}-{2\sigma }^{2}, h\right){I}_{{\varvec{q}}}$$

**Figure 3:**
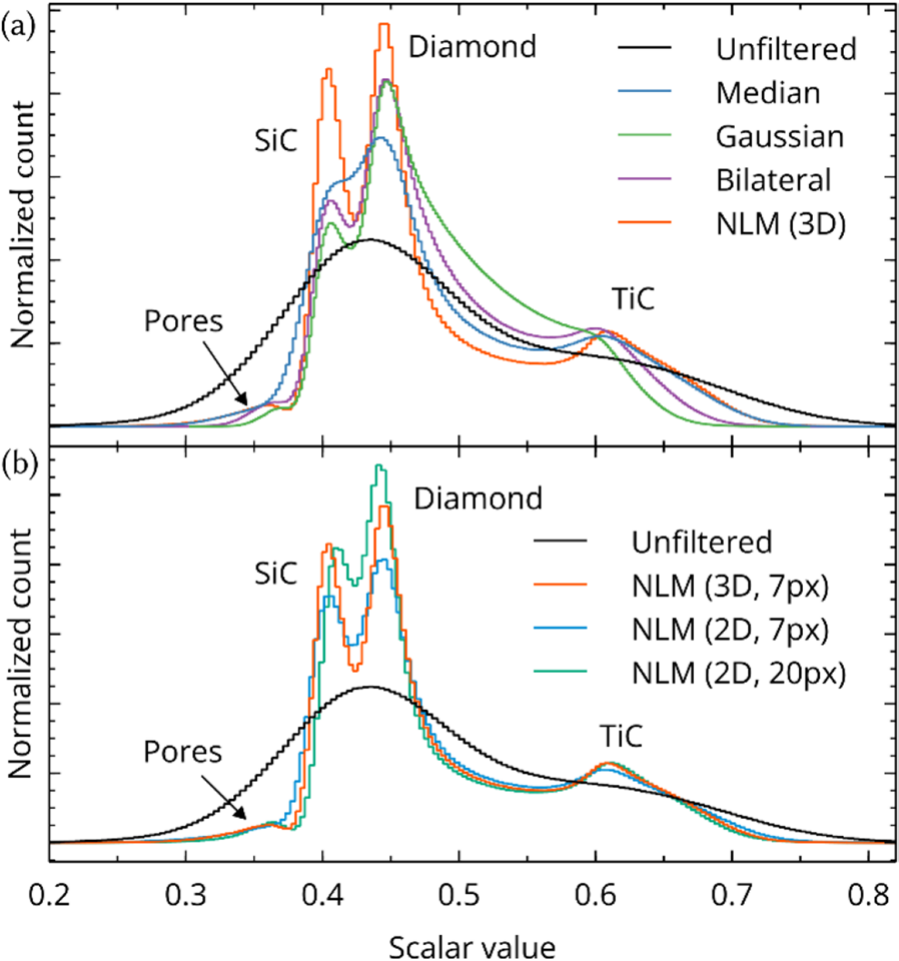
Scalar histograms of a SiC–TiC-diamond composite after various forms of denoising. (**a**) Median, gaussian, bilateral and 3D NLM denoising filters. (**b**) Comparison of 3D and 2D NLM filters. The parenthetic notation “NLM (A, B)” indicates the search dimensionality and search distance (A and B, respectively). The patch size, smoothing parameter, and noise estimate are constant for all spectra. Note that NLM (3D, 7px) and NLM (2D, 20px) incorporate a similar number of neighborhoods per pixel (343 and 400, respectively). All histograms are area-normalized to unity.

The ${\upsigma }^{2}$ value is an optional noise variance estimation, ensuring that any two neighborhoods within the noise variance are equally weighed (see^29^). The noise variance could be estimated by the median absolute deviation (MAD), wavelet shrinkage^30^, or any other number of estimators. The effect of each denoising filter on the scalar histogram, and an individual slice is shown in Fig. 3a and Fig. 2, respectively. In Fig. 3a, the visual quality of denoising is most evident by the distinctness of the SiC and diamond peaks. I.e., no material components of the unfiltered histogram are distinct (and the pores are entirely obfuscated). NLM gives the most visually definite peaks, due to both noise reduction and edge preservation.

While NLM is quite effective, its drawbacks come in the form of parameter estimation and computation time. While not exceedingly difficult, the patch size (size of ${{\varvec{\Delta}}}_{{\varvec{p}}}$ and ${{\varvec{\Delta}}}_{{\varvec{q}}}$), search neighborhood (size of $\Omega$), and smoothing parameter $h$ (standard deviation of ${s}_{\text{G}}$) must be manually estimated. The computational complexity of NLM is $O\left(N{P}^{d}{R}^{d}\right)$, where N is the total number of voxels, $P$ the patch size**,** $R$ the size of the search neighborhood, and $d$ the dimensionality. For our needs, we developed a fast multithreaded NLM implementation (C++ with a graphical user interface), with compiled binaries available^31^. However, those with access to Nvidia (or AMD ROCm-supported) graphics processing units (GPU) may find it prudent to use implementations eliciting GPU acceleration^18^. We created this software for quick, interactive parameter tuning and to lower the knowledge barrier for non-programmers.

Not all software supporting 3D NLM implement 3D neighborhoods/searching, many implementations accumulate 2D NLM filters in a paginated fashion over some axis of the 3D image. Paginated filtering will significantly reduce the computation time; however, the level of noise reduction will likewise diminish. For non-quantitative applications (i.e., XRM for purely imaging purposes), the speed gained by paginated filtering likely outweighs the boost in visual acuity. Figure 3b compares the effect of 3D NLM to 2D NLM for a SiC–TiC-diamond XRM composite. The distinctness of the SiC/Diamond peaks is best using 3D NLM; however, the other 2D NLM results are still quite favorable. For highly anisotropic microstructures, the denoising quality may be partially sensitive to the axis chosen for pagination. We find that the processing time for 3D NLM is approximately 10 × slower for the datasets and parameters used here. However, for quantitative applications—like porosity estimation—it may be prudent to favor denoising quality to minimize extraneous false positives/negatives generated during segmentation (i.e., see Fig. 7). The best choice likely depends on the data quality on a case-by-case basis, for this study we use 3D NLM for best quality denoising. There are further adaptations to the NLM filter to reduce execution time, based on Fast Fourier Transforms (FFT)^32^, omission of dissimilar neighborhoods^33^, probabilistic early termination^34^, or ^35^; all with varying levels of denoising quality.

### Segmentation challenges

Mapping the voxel-based representation of an XRM dataset into specific pore and non-pore (or pore and phasic) regions is the main pivotal step in porosity analysis. A robust method to classify regions of the dataset is critical, otherwise, phase fraction results become error-prone and reliant on manual intervention or correction by the individual. For well-behaved datasets, the segmentation step can be especially trivial if the density of each phase is well distributed. In these ideal cases, simple thresholding may be sufficient to wholly classify each phase. In this case, thresholding refers to segmentation by partitioning the dataset’s scalar distribution into fixed bins characterizing each phase. The “thresholds” define the boundaries of each bin.

**Figure 4:**
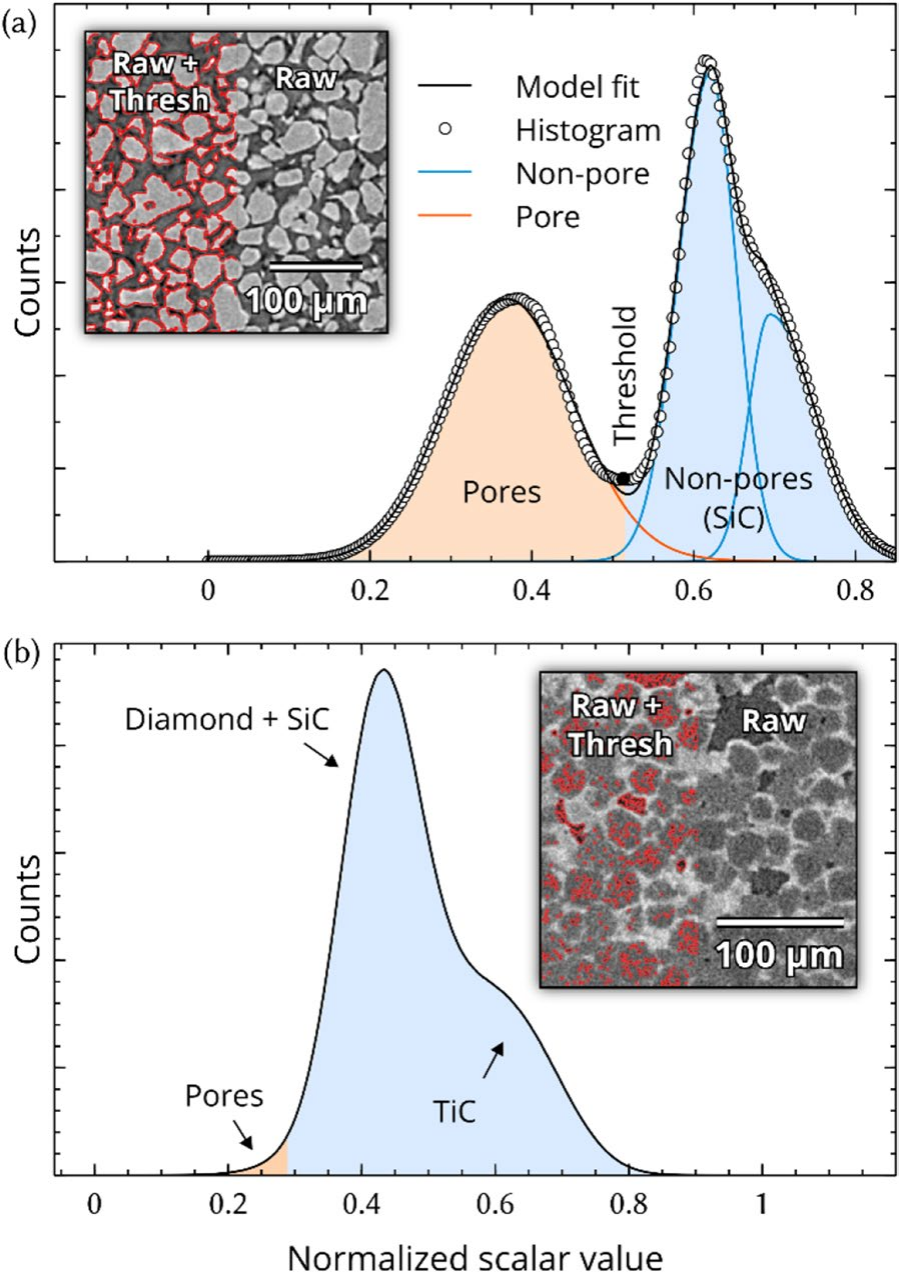
XRM histograms of (**a**) SiC and (**b**) SiC–TiC-diamond composite exhibiting isolated and obfuscated porosity peaks, respectively. (**a**) well-behaving histogram with isolated pore and non-pore peaks, a threshold position is set at ~ 0.52 with the resulting regions shaded in orange and blue. (**b**) poorly behaved histogram from a porous SiC–TiC-diamond composite containing 4 obscured phases; there is no obvious boundaries where thresholds should be placed to isolate pores (let alone any other phases).

The shaded regions in Fig. 4a show the result of thresholding on an intentionally porous SiC specimen (4 × binning), with respect to the dataset histogram and the inset image slice. There is a sufficient difference in density and recurrence among pores and SiC, such that peaks arising from pores are isolated from non-pores. Due to the peak isolation, a well-estimated threshold value could be placed around the valley at ~ 0.52, resulting in a porosity fraction of ~ 40.36 vol%. Non-linear least squares (NLLS) fitting of the histogram components is sometimes effective in estimating phase fractions, given a suitable model. Using a split-Voigt model for each peak results in a mediocre fit (orange for pores and blue for non-pores) and the porosity fraction is computed as the relative area of the pore model: ~ 42.92 vol% porosity. Still, there is a ~ 6% relative difference between the two methods arising from uncertainty in threshold placement and least squares parameter error (imperfect model). With such a high porosity content, this error is not too significant.

Figure 4b shows a similar histogram from an XRM experiment on a diamond-SiC–TiC composite (2 × binning). Unlike the porous SiC experiment, the diamond (~ 3.5 g/cm^3^), SiC (~ 3.16 g/cm^3^), and pore features are materially inseparable in their given state. The increase in noise is partially due to lower binning (needed to resolve smaller pores), causing the noise to increase by a factor of 8. Additionally, cutting diamond-based samples is non-trivial and can result in thicker than ideal XRM specimens, further increasing noise. Finally, the amount of porosity is much smaller (mostly < 6 vol%) resulting in a weaker, buried peak. Attempts at thresholding must be “eyeballed” to balance out the effects of erroneous false positives and negatives (see inset in Fig. 4b). Still, the result is inexact and difficult to visually confirm, depending on the accuracy needed. Further, we could not learn more about the pore distribution due to the heavily fragmented nature of the segmentation (i.e., what is the average pore size?). Also, without a physically meaningful model, NLLS deconvolution of the pore peak would likewise be hopeless.

Thresholding and NLLS fitting are not the only methods of isolating porosity information in tomographic datasets. Indeed, there are a multitude of algorithms for both instanced and semantic segmentation. These two methods, however, capture the essence of the ineffective separation of pores from non-pores in low-density ceramic materials (or otherwise noisy and low-contrast phases). The watershed algorithm—and its variations—may be the most traditional method used for segmentation in microscopy. If the dataset is thought of as a topographical map, the watershed treats the valleys as “basins” that fill with water. As the basins “fill” and begin to touch, the boundaries between basins can are retained. Resolving the boundaries (watersheds) between connected particles (basins) is the strength of watershed segmentation: improving particle analysis by the separation of contiguous particles that would otherwise be considered singular. For pore “particles”, however, there is not so much physical basis for the subdivision of contiguous pores.

### Application to porous SiC–TiC-diamond composites

**Figure 5:**
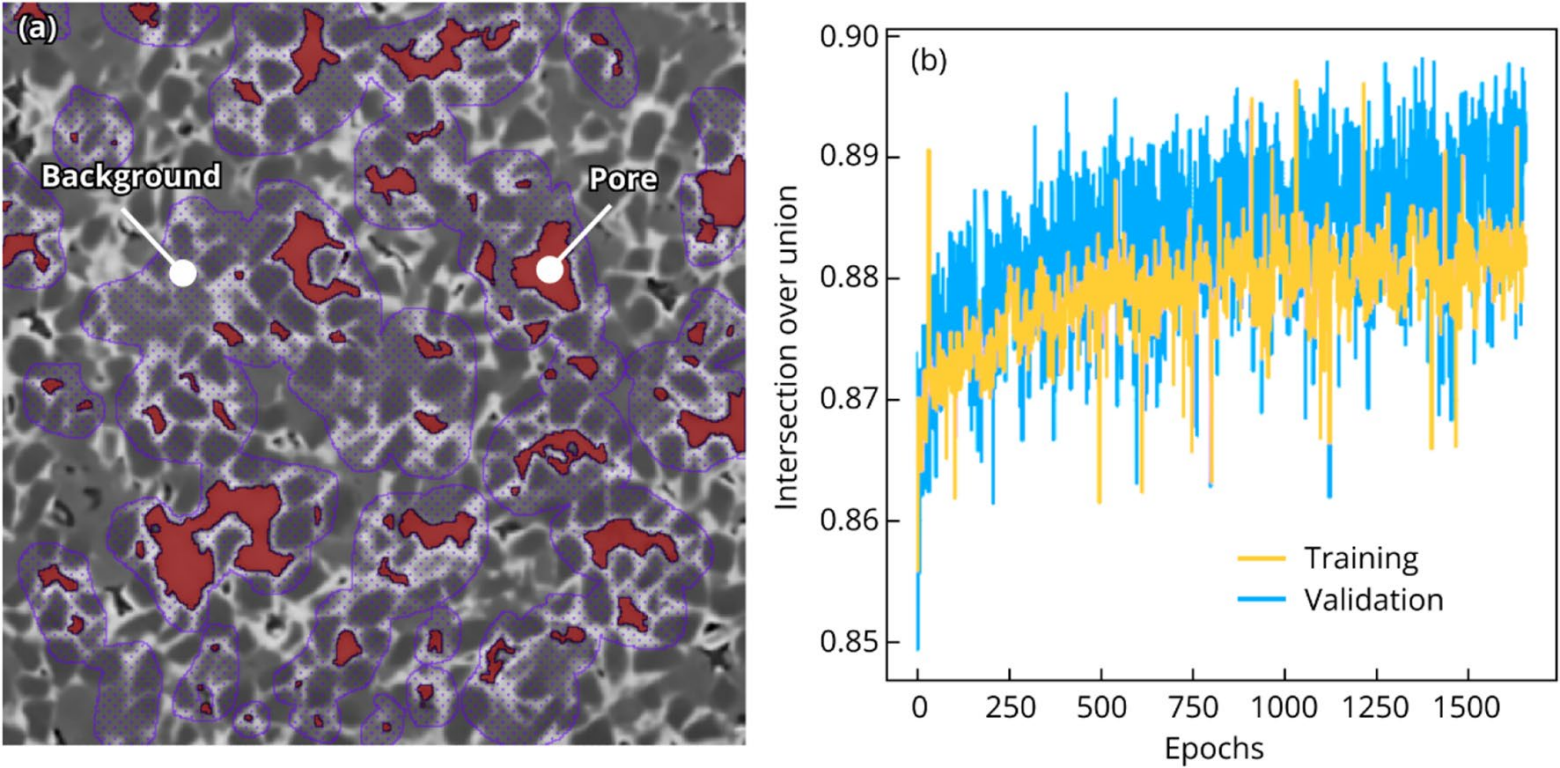
(**a**) Example annotated image for training. Pore annotations are represented in red and non-pore annotations (background) in purple. (**b**) Resulting intersection-over-union after model training.

Twenty slices from the 3 most porous composites were chosen for annotation and training. Chosen slices were well separated within each dataset, such that no pore was resampled by any other annotated slice. Pore boundaries among the 20 slices were carefully drawn for all sizes (small to large pores), then non-pore regions surrounding each pore were annotated (it is important to include pore boundaries in the background). An example slice is shown in Fig. 5a with annotated pore and background regions. Small, medium, and large pores are all represented in the annotation, and each pore boundary is included in the background regions. Every pore in a slice does not need to be annotated, we annotated ~ 80–90% of pores per slice. The composites are highly isotropic, and the axis chosen for annotation should not have an effect. The intersection-over-union (IoU) evolution of the model is shown in Fig. 5b with nominal IoU in the range 0.88–0.89.

**Figure 6:**
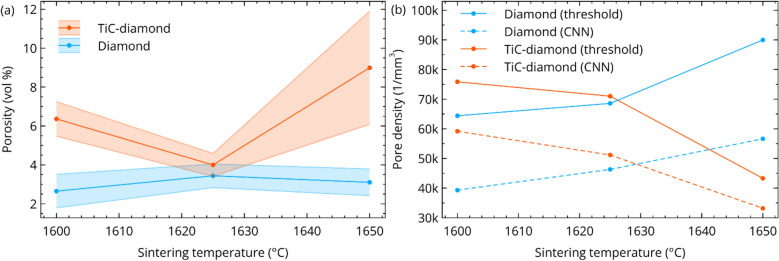
(**a**) Volume fraction porosity for six diamond-SiC–TiC (TiC-coated in orange, and uncoated in blue) composites, as determined by NLM-CNN processing. Error bars are the 95% confidence interval computed by analysis of the 3D pore distribution, see Eq. (7). (**b**) Pore number density as a function of temperature, compared by segmentation strategy.

Figure 6a shows the porosity fraction for the six composites resulting from the described analysis process, parameterized by the diamond powder (coated versus uncoated) and SPS hold temperature. The porosity of the uncoated diamond is constant with temperature when accounting for the estimated error. For TiC-coated diamond, the porosity is higher with a minimum at 1625 °C, where the porosity is in the vicinity of uncoated diamond. The higher porosity in TiC-coated diamond is attributed to delamination of the TiC coating as the underlying diamond surface graphitizes, resulting in a lower bulk density. Note that the size scale of graphite produced in this process (confirmed by transmission electron microscopy in^22^) is well below the detectable size for XRM.

**Figure 7:**
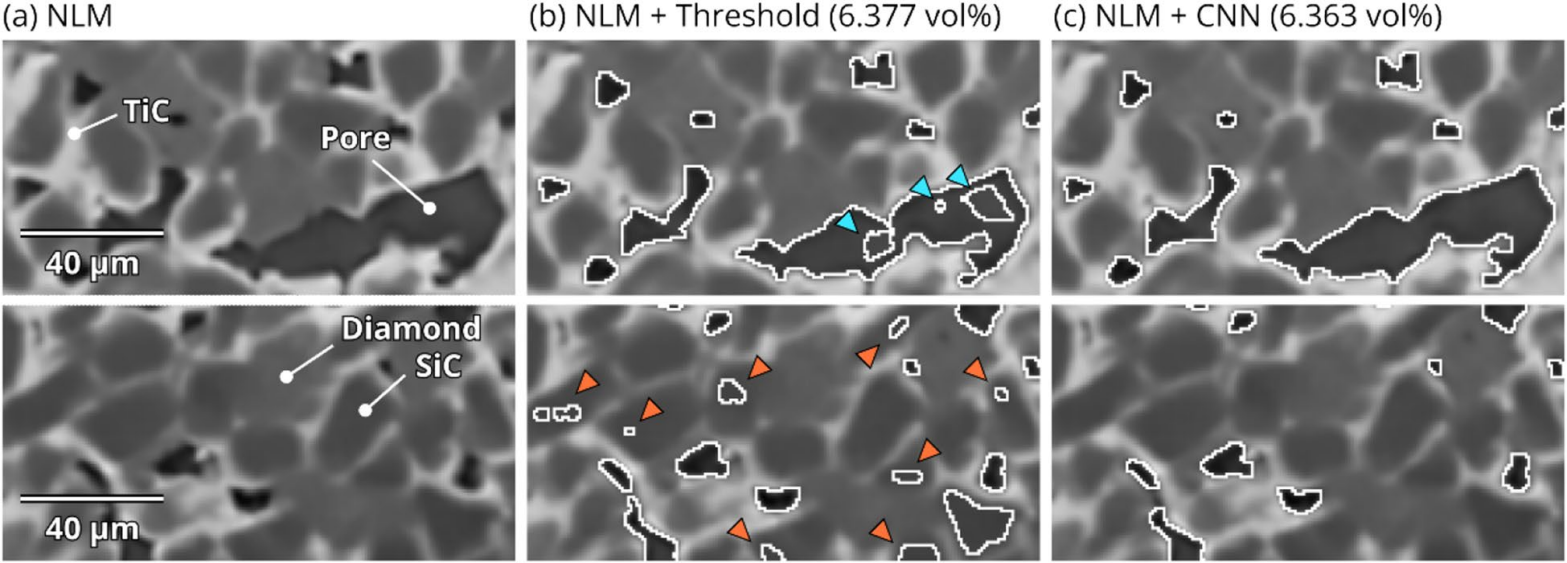
Visual evaluation of errors arising from segmentation. (**a**) Denoised and un-segmented areas from two regions in the 1600 °C uncoated diamond composite. (**b**) Threshold segmentation resulting in false negatives in the top area (blue) and false positives in the bottom (orange). (**c**) Convolutional neural network segmentation resulting in no false negatives/positives in the two regions. The threshold in (**b**) was chosen such that the resulting porosity is negligibly close to (**c**), the actual porosities are displayed above each image.

For statistics of the pore distribution to be viable, there must be a minimal amount of falsely classified pores (over/under-segmentation). Much of the potential for over/under-segmentation is mitigated by the denoising step (see the effect of noise on thresholding in Fig. 4). Elevated over-segmentation will result in elevated pore number density. Figure 6b conveys the difference in number density between thresholding and CNN segmentation, for each SiC–TiC-diamond composite. To make the comparison most sensible, the threshold value was chosen so the ensuing porosity fraction was equivalent to that measured by the CNN. Specifically, a scalar histogram of 1024 bins was computed for each dataset, and the bin value closest to the CNN porosity is chosen; the largest relative error was − 0.0083% in the 1650 °C TiC-coated diamond composite. In all cases, Fig. 6b consistently shows that the number density produced by CNN segmentation is consistently 20–40% lower than by thresholding, cursorily suggesting that over-segmentation is reduced using the CNN. To further explore whether this could be a result of under-segmentation of small pores by the CNN, Fig. 7 examines the visual effects of segmentation by both methods. For clarity, Fig. 7a shows the un-segmented regions. Figures 6c and 7b show the segmentation in equivalent regions (of the same slice) by thresholding and CNN segmentation, respectively. In the upper image, thresholding results in incomplete segmentation (false negatives) of the large pore, and over-segmentation (false positives) of diamond/SiC in the lower image. However, the CNN segmentation correctly classifies each image with neither over- nor under-segmentation.

### Estimation of uncertainty

Ideally, error estimations should accompany point estimates of the porosity fraction. An error estimate establishes a frame of reference for comparison among similar point estimates of different specimens or regions. The systematic error introduced by postprocessing XRM datasets through means of denoising or segmentation is not so straight forward to estimate. However, we can estimate the statistical error associated with the measured porosity distribution. I.e., the number, size, and spread of the pore distribution should affect our confidence in the point estimate being genuinely representative of the whole. Consider two specimens of equal porosity ${X}_{p}=50$ vol%: one containing a single enormous pore and the other thousands of finely dispersed and disconnected pores. In this example, we should have more confidence in the estimate containing numerous small pores, as opposed to that of a single large pore. In the contrived example of a single massive pore, we have little footing to expect whether a follow-up experiment would contain another pore, or two, or any at all. Moreover, we are confident that a follow-up experiment on the specimen with numerous finely dispersed pores should likely exhibit a similar distribution if the specimen is homogenous.

If the pore distribution is known, the porosity fraction can be expressed in terms of the mean pore volume $\overline{V}$, number of pores $n$, and the volume spanning the reconstruction $\Omega$:

4 $${X}_{p}=\frac{\overline{V}n}{\Omega }$$

Propagating the uncertainties of each variable approximates the standard error in ${X}_{p}$ as,

5 $$\delta {X}_{p}=\sqrt{{\left(\frac{\partial {X}_{p}}{\partial \overline{\mathrm{V}}}\right)}^{2}\delta {V}^{2}+{\left(\frac{\partial {X}_{p}}{\partial n}\right)}^{2}\delta {n}^{2}+{\left(\frac{\partial {X}_{p}}{\partial\Omega }\right)}^{2}\delta {\Omega }^{2}}$$

where the $\delta$ operator indicates the standard error of the mean. The reconstruction volume is exact, and the number of pores should be close to exact—provided the segmentation process does not generate needless false positives. If these assumptions hold, $\delta {X}_{p}$ becomes,

6 $$\updelta {X}_{p}=\frac{{X}_{p}}{\overline{V}}\updelta V$$

from which $\delta {X}_{p}$ and subsequent confidence intervals can be bootstrapped without further assumption. More concisely, if $\updelta V$ is taken as $\updelta V={s}_{V}/\sqrt{n}$, with ${s}_{V}$ being the standard deviation in pore volume, the standard error of the porosity fraction can be directly expressed as:

7 $$\updelta {X}_{p}=\frac{{X}_{p}{s}_{V}}{\overline{V}\sqrt{n}}$$

This procedure for estimating error should only apply to closed-cell type porosity. For open-cell porosity where a single pore could feasibly span the entire field of view, we cannot effectively sample the pore metrics, invalidating Eq. (6). In such circumstances, it may be prudent to pursue gaseous adsorption-based characterization vectors.

### Limitations

Compared to conventional denoising filters, non-local means requires a much longer execution time. The execution time can be considerably reduced by forgoing 3D neighborhoods in favor of 2D, at the slight expense of noise reduction (Fig. 3b). The reduced level of noise reduction may—or may not—be substantive when contrast is low. Parameter estimation may also require time if one is unfamiliar with NLM; this was partial motivation for the interactive NLM software we have provided.

Similarly, annotation and training of the neural network is a non-trivial time commitment, the number of annotations required is all but certain at the outset. With improvements like transfer learning^36^, or data augmentation such as elastic deformation fields^37^, the annotation commitment is being constantly reduced. Alternatively, annotation can be considered a tuning-knob of sort, as the segmentation quality may always be improved by simply more annotations—conventional segmentation methods rarely expose a vector for incremental improvement. However, the segmentation quality is not solely determined by the quantity of annotations, but by their quality too. Unfortunately, annotation is a human-driven process and is therefore captive to human bias.

## Conclusions

A novel combined post-processing methodology for quantitative porosity analysis is described within the context of X-Ray Microtomography. Comprised of three parts: (1) Signal/noise ratio enhancement using non-local means, (2) Pore segmentation using a convolutional neural network, and (3) Statistical analysis of the pore distribution, porosity metrics are robustly computed—despite elevated noise and low contrast exemplified by low-density ceramics. Porosity volume fraction estimates were computed for reactive spark plasma sintered SiC–TiC-diamond composites from 1600 to 1650 °C with, and without TiC coatings on the diamond particles (other porous low-density ceramics should work similarly well). The resulting TiC-coated diamond porosity was measured 4.0–9.0 vol%, and uncoated from 2.7 to 3.4 vol%, based on sintering temperature. Both over- and under-segmentation was significantly reduced by segmentation using a convolution neural network on non-local means pre-processed XRM datasets. The NLM-CNN method resulted in a 20–40% (based on temperature) reduction in estimated pores when compared to thresholding.

## Data Availability

All data generated or analyzed during this study are included in this published article.

## References

[1] Cuiyun D, Guang C, Xinbang X, Peisheng L (2012). Sound absorption characteristics of a high-temperature sintering porous ceramic material. Appl. Acoust..

[2] Du Z (2020). The sound absorption properties of highly porous silicon nitride ceramic foams. J. Alloys Compd..

[3] Hong W, Dong S, Hu P, Luo X, Du S (2017). In situ growth of one-dimensional nanowires on porous PDC–SiC/Si_3_N_4_ ceramics with excellent microwave absorption properties. Ceram. Int..

[4] Dong S, Zhang X, Zhang D, Sun B, Yan L, Luo X (2018). Strong effect of atmosphere on the microstructure and microwave absorption properties of porous SiC ceramics. J. Eur. Ceram. Soc..

[5] Ye F (2018). Direct growth of edge-rich graphene with tunable dielectric properties in porous Si_3_N_4_ ceramic for broadband high-performance microwave absorption. Adv. Funct. Mater..

[6] Jou CJ, Weber ER, Washburn J, Soffa WA (1988). Decoration of flux pinning positions in YBa_2_Cu_3_O_7-δ_ superconductors. Appl. Phys. Lett..

[7] Develos-Bagarinao K, Wimbush SC, Matsui H, Yamaguchi I, MacManus-Driscoll JL (2012). Enhanced flux pinning in MOD YBa_2_Cu_3_O_7−δ_ films by ion milling through anodic alumina templates. Supercond. Sci. Technol..

[8] Li D, Li M (2012). Porous Y_2_SiO_5_ ceramic with low thermal conductivity. J. Mater. Sci. Technol..

[9] Zhao N, Mao A, Shao Z, Bai H (2021). Anisotropic porous ceramic material with hierarchical architecture for thermal insulation. Bioinspir. Biomim..

[10] Klement U, Ekberg J, Kelly ST (2017). 3D analysis of porosity in a ceramic coating using X-ray microscopy. J. Therm. Spray Technol..

[11] O’Sullivan N, Mooney J, Tanner D (2021). Enhancing permeability and porosity of ceramic shells for investment casting through pre-wetting. J. Eur. Ceram. Soc..

[12] Klement U, Ekberg J, Creci S, Kelly ST (2018). Porosity measurements in suspension plasma sprayed YSZ coatings using NMR cryoporometry and X-ray microscopy. J. Coat. Technol. Res..

[13] Rubink WS (2021). Spark plasma sintering of B_4_C and B_4_C–TiB_2_ composites: Deformation and failure mechanisms under quasistatic and dynamic loading. J. Eur. Ceram. Soc..

[14] Fan L, Zhang F, Fan H, Zhang C (2019). Brief review of image denoising techniques. Vis. Comput. Ind. Biomed. Art.

[15] Mohan J, Krishnaveni V, Guo Y (2014). A survey on the magnetic resonance image denoising methods. Biomed. Signal Process. Control.

[16] Manjón JV, Carbonell-Caballero J, Lull JJ, García-Martí G, Martí-Bonmatí L, Robles M (2008). MRI denoising using non-local means. Med. Image Anal..

[17] Li A, Yu H, Gao J (2016). Probability-based non-local means filter for speckle noise suppression in optical coherence tomography images. Optics Lett..

[18] Roels J (2020). An interactive ImageJ plugin for semi-automated image denoising in electron microscopy. Nat. Commun..

[19] Kornilov AS, Safonov IV (2018). An overview of watershed algorithm implementations in open source libraries. J. Imaging.

[20] Yamashita R, Nishio M, Do RKG, Togashi K (2018). Convolutional neural networks: An overview and application in radiology. Insights Imaging.

[21] Haberl MG (2018). CDeep3M—Plug-and-play cloud-based deep learning for image segmentation. Nat. Methods.

[22] Garcia C, Smith JD, Rodriguez J, DiGiovanni AA, Scharf TW (2022). Reactive spark plasma sintering of SiC-TiC-diamond composites. Diam. Relat. Mater..

[23] Schindelin J (2012). Fiji: An open-source platform for biological-image analysis. Nat. Methods.

[24] Domander R, Felder AA, Doube M (2021). BoneJ2—Refactoring established research software. Wellcome Open Res..

[25] Tan, M., and Le, Q. V. EfficientNet: Rethinking model scaling for convolutional neural networks. Available: http://arxiv.org/abs/1905.11946 (2019).

[26] Aitken, A., Ledig, C., Theis, L., Caballero, J., Wang, Z., and Shi, W. Checkerboard artifact free sub-pixel convolution: A note on sub-pixel convolution, resize convolution and convolution resize. Available: http://arxiv.org/abs/1707.02937 (2017).

[27] Deng, J. Dong, W. Socher, R., Li, L.-J., Li, K., and Fei-Fei, L. ImageNet: A large-scale hierarchical image database. In: *2009 IEEE Conference on Computer Vision and Pattern Recognition*, 248–255 10.1109/CVPR.2009.5206848 (2009).

[28] Buades, A., Coll, B., and Morel, J. M. A non-local algorithm for image denoising. In: *Proceedings—2005 IEEE Computer Society Conference on Computer Vision and Pattern Recognition, CVPR 2005*, Vol. II 60–65 10.1109/CVPR.2005.38 (2005).

[29] Buades A, Coll B, Morel J-M (2011). Non-local means denoising. Image Process. Line.

[30] Donoho DL, Johnstone IM (1994). Ideal spatial adaptation by wavelet shrinkage. Biometrika.

[31] Smith, J. D. Non-local means denoiser. https://gitlab.com/jesseds/nlm.

[32] Liu YL, Wang J, Chen X, Guo YW, Peng QS (2008). A robust and fast non-local means algorithm for image denoising. J. Comput. Sci. Technol..

[33] Mahmoudi M, Sapiro G (2005). Fast image and video denoising via nonlocal means of similar neighborhoods. IEEE Signal Process. Lett..

[34] Vignesh R, Oh BT, Kuo CCJ (2010). Fast non-local means (NLM) computation with probabilistic early termination. IEEE Signal Process. Lett..

[35] Darbon, J., Cunha, A., Chan, T. F., Osher, S., and Jensen, G. J. Fast nonlocal filtering applied to electron cryomicroscopy. In: *2008 5th IEEE International Symposium on Biomedical Imaging: From Nano to Macro, Proceedings, ISBI* 1331–1334. 10.1109/ISBI.2008.4541250 (2008).

[36] Zhuang F (2021). A comprehensive survey on transfer learning. Proc. IEEE.

[37] Chaitanya, K., Karani, N., Baumgartner, C. F., Becker, A., Donati, O., and Konukoglu, E. Semi-supervised and task-driven data augmentation. In: *Lecture Notes in Computer Science (including subseries Lecture Notes in Artificial Intelligence and Lecture Notes in Bioinformatics) LNCS*, Vol. 11492 29–41 10.1007/978-3-030-20351-1_3/FIGURES/3 (2019).

